# Effects of white mulberry powder fortification on antioxidant activity, physicochemical, microbial and sensorial properties of yogurt produced from buffalo milk

**DOI:** 10.1002/fsn3.3053

**Published:** 2022-09-15

**Authors:** Sania Sheikh, Farzana Siddique, Kashif Ameer, Rabia Shabbir Ahmad, Aneela Hameed, Asma Ebad, Isam A. Mohamed Ahmed, Sahar Shibli

**Affiliations:** ^1^ Institute of Food Science and Nutrition University of Sargodha Sargodha Pakistan; ^2^ Department of Food Science Government College University Faisalabad Faisalabad Pakistan; ^3^ Institute of Food Science & Nutrition Bahauddin Zakariya University Multan Pakistan; ^4^ Department of Food Science and Technology, Faculty of Agriculture University of Khartoum Shambat Sudan; ^5^ Department of Food Science and Nutrition, College of Food and Agricultural Sciences King Saud University Riyadh Saudi Arabia; ^6^ Food Science Research Institute National Agricultural Research Centre Islamabad Pakistan

**Keywords:** antioxidant potential, physicochemical analysis, sensory evaluation, sun‐dried white mulberry powder, yogurt

## Abstract

The objective of this study was to investigate the effects of sun‐dried white mulberry fruit powder (WMFP) at addition levels of 2%, 4%, and 6% for enhancing nutritional value and improving the quality of yogurt during refrigerated storage. Results showed that the highest (*p* < .05) antioxidant activity of 54.53 **±** 0.15% was observed in 6% WMFP‐added yogurt samples. Highest pH of 4.53 ± 0.08 was observed in control. Significantly highest (*p* < .05) acidity (1.12 ± 0.02%) was recorded in the yogurt with 6% WMFP‐added yogurt samples on 28th day. Moreover, the addition of WMFP elevated the total soluble solids up to 20.05 ± 0.02 °Brix and water‐holding capacity up to 55.06 ± 0.34% and lessened the syneresis value (22.92 ± 0.25**)** in 6% yogurt samples. 2% WMFP‐added yogurt sample was given the highest sensory score in terms of overall acceptability by the panelists (8.00 ± 0.00). Thus, it was concluded that fruit powder of white mulberry can be used to improve physicochemical and sensory properties of fortified yogurt. WMFP addition to yogurt enhanced its antioxidant potential and physicochemical quality. The study introduces white mulberry‐enriched yogurt and suggests the food industries to launch this product in the market.

## Graphical abstract

Fruit powder of white mulberry was used to improve physicochemical and sensory properties of fortified yogurt. This addition in yogurt enhanced its antioxidant potential and physicochemical quality. The study introduces white mulberry enriched yogurt and suggests the food industries to launch this product in the market.

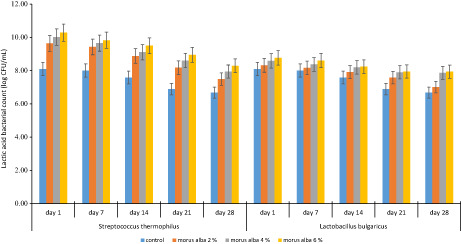

## 1 INTRODUCTION

Mulberry is a plant belonging to the genus *Morus* in family *Moraceae* and is widely available in various regions around the globe specifically ranging from temperate to tropical. The term “*Mora*” is thought to be originated from the Latin word “*Mora*” which literally means delay, and this name was denoted because of likely slow development of buds in plants of genus *Morus*. Commonly, white mulberry (*Morus alba*) is one of the dominant species (Jan et al., 2021). Almost all mulberry varieties have been recorded in traditional Chinese, Ayurvedic, Unani systems of medicines with several important pharmacological properties. In India and Pakistan, mulberry plant is commonly known in masses with the names of KalpaVruksha and Shahtoot (meaning superior mulberry), respectively, as all parts of mulberry plant are utilized for various purposes (Hashemi & Khadivi, 2020; Sharma et al., 2020). Its leaves, roots, bark, stems, twigs, and fruit exhibit significant therapeutic significance owing to the presence of bioactive constituents which may be exploited in formulation of food, healthcare, and cosmetic products. Some major bioactive components of mulberry include organic sugars (glucose and fructose), organic acids (malic, succinic, and fumaric acids), tannins, and anthocyanins. Other bioactive phytoconstituents include triterpenes, tannins, phytosterols, sitosterols, saponins, flavonoids, morusimic acid, benzofuran derivatives, anthocyanins, glycosides, anthraquinones, and oleanolic acid (Chen et al., 2005; Neamat‐Allah et al., 2021). In addition to this, it also contains vitamin C and minerals like calcium and potassium. In Chinese Pharmacopeia, mulberry fruit is recommended to treat various ailments as natural remedy inducing, joints pain, hypertension, fever, liver repair, and as diuretic for urine discharge (Sung et al., 2015). In traditional Turkish folk medicines, the mulberry fruit, leaves, and bark are employed for fever and diabetes treatment, lowering blood pressure, treating dental issues, arthritis, and anemia, as deworming agent, laxative, and odontalgic agent (Gryn‐Rynko et al., 2016). The presence of valuable bioactive compounds in mulberry makes it an ideal candidate in functional foods category for promoting human health in addition to fulfillment of nutritional requirement (Butkhup et al., 2013).

Yogurt is an ancient dairy product obtained by lactic acid fermentation involving the action of *Lactobacillus bulgaricus* and *Streptococcus thermophilus* (El‐Saadony et al., 2021). Yogurt is rich in nutrients like potassium, calcium, protein, and B complex vitamins. Above all, yogurt has probiotic properties making yogurt an ideal candidate with health‐promoting effects on digestion, cholesterol reduction, and treating diarrhea. Moreover, yogurt has been appraised by the consumers worldwide as the most popular and in‐demand fermented dairy product not only because of its nutritional profile but for its health‐promoting benefits as well (Savaiano & Hutkins, 2021).

Fruits, nuts, vanilla, and coffee are the flavors commonly added to yogurt. The most popular and appealed yogurt flavor among the consumers till date is strawberry flavor (Lim et al., 2019). Although dairy products have their obvious health benefits, however, fermented dairy products, such as yogurt and curd, are not documented as rich source of bioactive compounds like antioxidants and polyphenols. This necessitates to formulate the novel dairy products by supplementing and fortifying with medicinal herbs or phytochemical‐rich plant extracts to satisfy the need of health‐conscious consumers. In order to exploit the nutraceutical potential of mulberry, possibility of mulberry introduction as functional food and nutraceutical is need of the time by supplementation/fortification to improve the storage stability, physicochemical, and organoleptic properties of dairy products like yogurt. To best of our knowledge, yogurt added with white mulberry has never been studies so far. Therefore, the objective of this study was to investigate the possible use of sun‐dried white mulberry fruit powder (WMFP) for enhancing nutritional value and improving the quality of yogurt during refrigerated storage and evaluation of effects of WMFP on antioxidant activity, lactic acid bacterial count, physicochemical and sensory quality of yogurt.

## 2 MATERIALS AND METHODS

### 2.1 Sample procurement and preparation

White mulberry fruit was procured from the local market of Sargodha, Pakistan. The mulberry fruits were subjected to water washing followed by sun‐drying at an ambient temperature (30°C). The sun‐dried mulberry fruits were disintegrated into small pieces and then ground using lab‐scale grinder to prepare fruit powder (WMFP). Sieving with 60‐mesh sieve was carried out to achieve the uniform particle size. Then, the WMFP was packed in the polythene bags, stored at freezing temperature (−18°C), and was employed in yogurt processing during storage study period.

All solvents employed in this study were of analytical grade. The reagent 2, 2‐diphenyl‐1‐picrylhydrazyl (DPPH), Folin–Ciocalteu, and gallic acid were obtained from Sigma‐Aldrich.

### 2.2 Proximate analysis of mulberry fruit powder

WMFP was subjected to proximate analysis as per the methods described in AOAC. WMFP was analyzed for its moisture, ash, and crude fiber through the gravimetric method as described in AOAC method No. 934.01, dry incineration in muffle furnace according to AOAC method No. 976.05 and Soxhlet method (AOAC method No. 954.02), respectively. Kjeldahl method was used for the determination of the protein content according to the AOAC method No. 976.05. The obtained results were indicative of the total nitrogen content which were further multiplied by the factor 6.25 for total protein content determination. Difference method was employed for the determination of the total carbohydrate content as per the following Equation 1:

(1) $$\text{Total carbohydrate}\;\left(\%\right)=\left(100-\left[\text{Moisture}+\text{Protein}+\mathrm{Fat}+\mathrm{Ash}\right]\right).$$

TPC and TFC of WMFP were determined spectrophotometrically at 765 and 510 nm, respectively, according to the methods described by Jiang et al. (2021). The TPC was expressed as gallic acid equivalents (mg GAE 100 g^−1^) on dry weight (DW) basis, whereas TFC results were represented as milligram rutin equivalent (mg RE 100 g^−1^) on DW basis.

### 2.3 Preparation of yogurt

Sun‐dried white mulberry fruit powder (2%, 4% and 6%) was added in buffalo milk having 4.46% protein, 7.2% fat, 0.81% ash, 16.73% total soluble solids (TSS), and blended with lab‐scale blender for 2 min. When complete solubilization of sun‐dried WMFP in the buffalo milk was achieved, then the milk was pasteurized at 90°C for 10 min in a water bath. After cooling, Direct Vat Set (DVS) culture (ThermophilicYoFlex starter) consisting of *Lactobacillus bulgaricus* and *Streptococcus thermophilus* (Chr. Hansen) (2%) was added and was left for incubation at 42 ± 1°C. After reaching a pH of 4.6, all yogurt samples were kept at 5°C for 28 days. All the experiments were carried out in triplicate (*n* = 3).

### 2.4 Determination of antioxidant activity

Antioxidant potential of WMFP‐added yogurt samples was measured according to the method described by Blois, 1958. Homogenization of 1 g yogurt with 10 ml of ethanol was done. 0.2 ml of the mentioned solution was mixed with 0.8 ml of DPPH solution (1.5 × 10^−4^ M) and was allowed to stand for 30 min at a place where no light could approach it. Absorbance was measured by UV spectrophotometer (UV‐1800; Shimadzu Instruments Mfg. Co., Ltd.) at 517 nm against a blank. All measurements were performed in triplicate. The degree of discoloration was measured by the following equation:

(2) DPPH activity%=AbsControl−AbsSampleAbsControl×100.

Abs control represents the absorbance of DPPH radical with ethanol and Abs sample symbolizes the absorbance of DPPH radical with yogurt samples.

### 2.5 Physicochemical analysis

#### 2.5.1 pH and acidity

pH meter (pH 211 Microprocessor pH meter; Hanna Instruments) was used to measure the pH values of the samples. Yogurt was homogenized in water (1:9 ratio) preceding to pH determination as per AOAC method 943.02 (AOAC, 2000). The titratable acidity was determined by titration using a 0.1 N sodium hydroxide (NaOH) solution to the endpoint of pink color.

#### 2.5.2 Total soluble solids

Yogurt (2 g) was placed in a hot air oven for 3 h at 100°C and then cooled in a desiccator for 30 min. The percentage of residues obtained was the total soluble solids (TSS) (AOAC, 2000).

(3) TSS°Brix=Weight of sample after dryingWeight of sample before drying×100.

#### 2.5.3 Water‐holding capacity (WHC)

The centrifugation of 20 g yogurt sample at 669× *g* for 10 min was carried out and the supernatant was removed and weighed (Arslan & Ozel, 2012).

(4) WHC%=Weight of Sample−Whey expelledWeight of Sample×100.

#### 2.5.4 Syneresis

A 200 g of each of yogurt samples (*Y*) was taken and centrifuged at 4°C for 10 min at 770× *g*. The syneresis was measured as per the reported method of Abd El‐Salam et al. (1991).

(5) Syneresis%=Totalwt.of separated liquidgTotalwt.of yogurt sampleg×100.

### 2.6 Lactic acid bacteria count

Yogurt samples were diluted by serial dilution with peptone water. 1 ml of each diluted sample was plated on MRS (De Man, Rogosa, Sharpe) (Dehydrated) and M17 Agar (Dehydrated) media (Thermo Fisher Scientific) according to pour‐plate method used for the enumeration of *L. bulgaricus* and *S. thermophilus*, respectively. The plates were incubated at 37°C for 72 h and at 42°C for 48 h for enumeration of *L. bulgaricus* and *S. thermophilus*, respectively. The colonies formed were counted and expressed as log CFU/ml (Ogunsina et al., 2011).

### 2.7 Sensory evaluation

Sensory evaluation was carried out by the 20 semi‐trained panelists in laboratory using a 9‐point hedonic scale with following score expressions: 9 = like very much, 8 = like a lot, 7 = rather like, 6 = quite like, 5 = neither like nor dislike, 4 = dislike a little, 3 = rather dislike, 2 = dislike very much, 1 = highly dislike. Different sensory attributes were evaluated, such as color, flavor, texture, sweetness, sourness, taste, and overall acceptance. All individuals were instructed well before analysis. All measurement were recorded in triplicate (*n* = 3).

### 2.8 Statistical analysis

Results obtained from different parameters were subjected to statistical analysis using one‐way analysis of variance (ANOVA) technique. The differences between the means were determined through Duncan multiple range test at significance level of *p* < .05 (Steel, 1997).

## 3 RESULTS AND DISCUSSION

Nutritional composition (protein, fat, crude fiber, ash, and carbohydrate) in terms of g/100 g on dry weight (DW) basis, total phenolic content (TPC: mg GAE/g DW), antioxidant activity (AOA %), and flavonoid content of WMFP is provided in Table 1. The moisture, protein, fat, crude fiber, ash, and carbohydrates contents of WMFP were found to be 83.76, 1.65, 0.49, 1.39, 0.57, and 12.62%, respectively. Whereas total phenolic content, antioxidant activity, and flavonoid content were found to be 13.82 mg GAE/g DW, 31.96%, and 4.72 mg/g (DW basis), respectively.

**TABLE 1:** Nutritional composition (g/100 g DW), TPC (total phenolic content mg GAE/g DW), and antioxidant activity (AOA %) of white mulberry

Parameters	Content g/100 g (DW basis)
Moisture	83.76 ± 1.59
Protein	1.65 ± 0.068
Fat	0.49 ± 0.035
Crude fiber	1.39 ± 0.06
Ash	0.57 ± 0.053
Carbohydrates	12.62 ± 0.1
TPC	13.82 ± 0.65 mg GAE/g DW
Antioxidant activity	31.96 ± 0.57%
Flavonoids	4.72 ± 0.23 mg RE/g DW

### 3.1 Effect on antioxidant activity (DPPH %) of yogurt

The results of effect of WMP concentration and storage period on antioxidant activity (DPPH %) of yogurt are given in Table 2. Antioxidant activity of control (*T*_0_) samples during all storage periods ranged from 26.43% to 33.37%. Antioxidant activity of 2% WMFP‐supplemented samples (*T*_1_) during all storage periods (1–28 days) ranged from 33.73% to 42.51%. In case of 4% WMFP‐supplemented yogurt samples, the antioxidant activity was found to be in range of 43.41%–51.57% during all storage intervals (1–28 days). In case of 6% WMFP‐supplemented yogurt samples, the antioxidant activity was found to be in range of 51.50%–57.59% during all storage intervals ranging from 1 to 28 days. Overall, it was evident from the results that all WMFP‐supplemented samples showed increasing tendencies in concentration‐dependent manner regarding antioxidant activity during all storage intervals. At day 1, the highest antioxidant potential was observed in yogurt added with 6% WMFP and recorded activity was found to be 54.63%, whereas 2% supplemented samples exhibited the lowest antioxidant activity (37.41%) in comparison with control (30.31%) (Table 2). On day 7, the antioxidant activity showed rises in gradual manner regardless of the sample type. Highest antioxidant activity was exhibited by 6% supplemented (*T*_3_) yogurt samples. On days 14, 21, and 28, all samples including control exhibited the gradually declining trends in antioxidant activity. On day 28, the 6% supplemented samples had the highest antioxidant activity (51.50%) as compared to 4% (43.41%), 2% supplemented (33.73%), and control (26.43%) yogurt samples. Increase in antioxidant potential with the addition of WMFP might be due to the presence of abundant phytochemicals in white mulberry. Some of the antioxidant compounds reported to be in white mulberry are ascorbic acid, β‐carotene, vitamin B1, folic acid, folinic acid, isoquercetin, quercetin, tannins, flavonoids, and saponins (Devi et al., 2013). The increase during storage may be attributed to the metabolic activity of LAB that remains active during refrigerated storage (Hammon & Blum, 1998). Increased antioxidant activity in fermented milk products is due to the proteolysis by lactic acid bacteria and the subsequent release of bioactive peptides (Pihlanto, 2006). These results were in agreement with the findings of Sung et al. (2015) who reported increases in antioxidant activity of fortified yogurt because of the presence of tyrosine amino acid in fortified yogurt composition as the amino acid tyrosine has been reported to exhibit phenolic side chain in its configuration (Chen et al., 2006). Moreover, the highest antioxidant potential of supplemented yogurts might be attributable to the increased anthocyanin content as anthocyanins are group of compounds which have contributory role in imparting of color to the fruits, plants, and flowers (Thompson et al., 2007). During storage, the 6% supplemented yogurt samples did not exhibit any significant decrease in antioxidant activity which implied that antioxidant compounds in yogurt remained stable with slight changes in decreasing manner owing to anthocyanins stability in supplemented yogurt samples as well as compared to control. Moreover, the higher antioxidant activity of supplemented yogurt samples was attributed to the individual *M. alba* phytochemical contents and microbial metabolic activities during growth phases of mulberry plant (Thompson et al., 2007). During refrigerated stoppage, the decreasing trend in antioxidant activity of yogurt samples could be ascribed to the fact of phenolic compounds degradation or increased formation of complex by interaction of polyphenols with milk proteins (Yuksel et al., 2010).

**TABLE 2:** Effect of white mulberry powder concentration and storage period on antioxidant activity (DPPH %) of yogurt

Storage period (day)	*T*_0_ (control)	*T*_1_ (2%)	*T*_2_ (4%)	*T*_3_ (6%)
1	30.30 ± 0.23°	37.40 ± 0.15^l^	46.63 ± 0.18^g^	54.63 ± 0.09^c^
7	33.37 ± 0.12^n^	42.50 ± 0.15^j^	51.57 ± 0.19^e^	57.57 ± 0.19^a^
14	29.53 ± 0.12^p^	38.43 ± 0.22^k^	49.37 ± 0.12^f^	55.37 ± 0.12^b^
21	27.40 ± 0.17^q^	34.53 ± 0.12^m^	45.60 ± 0.15^h^	53.60 ± 0.21^d^
28	26.43 ± 0.19^r^	33.73 ± 0.09^n^	43.40 ± 0.20^i^	51.50 ± 0.15^e^

*Notes*: The antioxidant content was measured in %. Mean values are the result of three replications (*n* = 3). Means carrying the same small letters are significantly different from each other. *T*_0_: Control; T_*1*_: Sample fortified with 2% white mulberry powder concentration; *T*_2_: Sample fortified with 4% white mulberry powder concentration; *T*_3_: Sample fortified with 6% white mulberry powder concentration.

### 3.2 Effect on pH, titratable acidity, and TSS of yogurt samples

The results of WMFP‐supplemented and control yogurt samples are given in Table 3 during storage period ranging from 1 to 28 days. As evident from results of Table 3, pH of control (*T*_0_) samples during all storage periods were ranged from 4.41 to 4.69. The pH values of 2% WMFP‐supplemented samples (*T*_1_) subjected to storage period from 1 to 28 days were found in range of 4.18–4.55. In case of 4% WMFP‐supplemented yogurt samples, the pH was found to be in range of 3.97–4.49 during all storage intervals spanning 1–28 days. In case of 6% WMFP‐supplemented yogurt samples, the pH range was 3.67–4.28 during all storage intervals. On day1, the pH ranged 4.28–4.69. pH was found highest (4.69 ± 0.15) in control yogurt at day 1 while the lowest pH (3.66 ± 0.02) was observed at day 28 in the yogurt having 6% concentration of a WMFP. Concentration‐dependent decreases were evident in supplemented yogurt samples with corresponding rises in addition levels of mulberry fruit powder. Similarly, the pH ranges on day 7, 14, and 21 were 4.17–4.58, 4.08–4.53, 3.85–4.45, respectively and all supplemented samples exhibited slightly decreasing tendencies in pH values with corresponding increases in WMFP addition levels from 2% to 6%. Similar, as far as the storage period effect is concerned, the yogurt samples including control showed gradually decreasing tendencies with increases in storage period from 7 to 28 days. In case of 2% supplemented samples, the pH decrease was not so much significant (*p* > .05) as compared to control whereas 4% and 6% supplemented samples exhibited significantly lower (*p* < .05) pH values on days 21 and 28 in comparison with control. During storage, the decreasing tendency in pH values of supplemented and control yogurt samples could be attributed to the fact of lactic acid neutralization because of mulberry powder addition (Sung et al., 2015). Acid development in slow manner is undesirable property for yogurt development; therefore, increased acidity in supplemented yogurts was evident of the fact that WMFP caused stimulation of whey separation and symbiotic relationship breakdown among started bacteria (Celik & Bakirci, 2003). The decrease in pH is indicative of rises in acidity levels, and hence, the results corroborated with the decreasing pH tendencies with corresponding rises in storage period and WMFP addition levels from 2% to 6%. As evident from results of Table 3, titratable acidity of control (*T*_0_) samples during all storage periods was ranged from 0.90% to 1.05%. Titratable acidity of 2% WMFP‐supplemented samples (*T*_1_) subjected to storage period from 1 to 28 days was found in range of 0.92%–1.07%. In case of 4% WMFP‐supplemented yogurt samples, the titratable acidity was found to be in range of 0.95%–1.09% during all storage intervals spanning 1–28 days. In case of 6% WMFP‐supplemented yogurt samples, the titratable acidity was 0.98–1.12 during all storage intervals. Overall, it was observed that titratable acidity exhibited concentration‐dependent rises. For same sample, the increasing trend was evident with increases in storage interval from 1 to 28 days. On 28th day, significantly highest acidity (1.12 ± 0.02%) was recorded in the yogurt with 6% WMFP, while lowest acidity (0.90 ± 0.01%) was found in control yogurt at the beginning of experimentation (Table 3). For all samples including control, the titratable acidity levels on day 1, 7, 14, and 28 were found to be in ranges of 0.90%–0.98%, 0.92%–0.99%, 0.95%–1.03%, 0.102%–1.08%, and 1.05%–1.12%, respectively. In a similar study conducted by Lee and Lee (2014), pH was found that 20% supplemented yogurt samples with barley flour exhibited higher acidity values as compared to that of control (un‐supplemented) samples. Titratable acidity of yogurt samples enhanced owing to the presence of organic acids in composition of white mulberry fruit powder. In this regard, notable organic acids possibly may include butyric, acetic, formic, citric, and lactic acids. These results are in agreement to the previous studies where authors suggested that white mulberry has the general effect to lessen the pH of yogurt (Mahmood et al., 2008; Tarakci et al., 2013). The increase in acidity may be attributed to the growth of lactic acid bacteria (Servili et al., 2011).

**TABLE 3:** Effect of white mulberry powder concentration and storage period on pH, titratable acidity, and total solids of yogurt

Parameters	Storage period (day)	*T*_0_ (control)	*T*_1_ (2%)	*T*_2_ (4%)	*T*_3_ (6%)
pH	1	4.69 ± 0.15^a^	4.55 ± 0.13^abc^	4.49 ± 0.09^bcd^	4.28 ± 0.04^efg^
	7	4.58 ± 0.12^ab^	4.51 ± 0.06^abc^	4.38 ± 0.03^cde^	4.17 ± 0.02^fgh^
	14	4.53 ± 0.10^abc^	4.41 ± 0.03^bcde^	4.26 ± 0.02^efgh^	4.08 ± 0.04^hi^
	21	4.44 ± 0.02^bcde^	4.32 ± 0.03^def^	4.13 ± 0.02^ghi^	3.85 ± 0.03^j^
	28	4.40 ± 0.01^cde^	4.18 ± 0.04^fgh^	3.97 ± 0.04^ij^	3.66 ± 0.02^k^
Titratable acidity (%)	1	0.90 ± 0.01^j^	0.92 ± 0.01^ij^	0.95 ± 0.02^gh^	0.98 ± 0.01^fg^
	7	0.92 ± 0.01^ij^	0.94 ± 0.01^hi^	0.96 ± 0.01^gh^	0.99 ± 0.00^f^
	14	0.95 ± 0.00^gh^	0.97 ± 0.01^fgh^	0.99 ± 0.01^f^	1.03 ± 0.01^de^
	21	1.02 ± 0.01^e^	1.03 ± 0.00^de^	1.05 ± 0.00^cd^	1.08 ± 0.01^bc^
	28	1.05 ± 0.01^cd^	1.07 ± 0.00^bc^	1.09 ± 0.00^b^	1.12 ± 0.02^a^
Total soluble solid (%)	1	15.29 ± 0.02^p^	16.87 ± 0.08^k^	18.83 ± 0.03^f^	20.53 ± 0.03^a^
	7	15.17 ± 0.01^q^	16.74 ± 0.02^l^	18.61 ± 0.05^g^	20.25 ± 0.02^b^
	14	15.08 ± 0.01^r^	16.39 ± 0.05^m^	18.22 ± 0.03^h^	20.04 ± 0.02^c^
	21	14.94 ± 0.01^s^	16.19 ± 0.01^n^	18.05 ± 0.02^i^	19.86 ± 0.02^d^
	28	14.81 ± 0.01^t^	15.78 ± 0.04°	17.81 ± 0.01^j^	19.56 ± 0.02^e^

*Notes*: Mean values are the result of three replications (*n* = 3). Means carrying the same small letters are significantly different from each other. *T*_0_: Control; *T*_1_: Sample fortified with 2% white mulberry powder concentration; *T*_2_: Sample fortified with 4% white mulberry powder concentration; *T*_3_: Sample fortified with 6% white mulberry powder concentration.

As evident from results of Table 3, TSS of control (*T*_0_) samples during all storage periods (from 1 to 28 days) were ranged from 14.81% to 15.29%. TSS of 2% WMFP‐supplemented samples (*T*_1_) subjected to storage period from 1 to 28 days were found in range of 15.78%–16.87%. In case of 4% WMFP‐supplemented yogurt samples, the TSS was found to be in range of 17.81%–18.83% during all storage intervals spanning 1–28 days. In case of 6% WMFP‐supplemented yogurt samples, the TSS content range was 19.56%–20.53% during all storage intervals. Overall, the TSS content increased with an increase in the concentration of white mulberry fruit powder and decreased with the increase in the number of days (Table 3). Addition of slight amounts of WMFP significantly resulted in increased TSS. The TSS content on day 1, 7, 14, 21, and 28 was recorded in ranges of 15.29–20.53, 15.17–20.25, 15.08–20.04, 14.94–19.86, and 14.81–19.56 °Brix, respectively. Until day 21, the TSS content exhibited very slight decreases, however, on day 28, the TSS content decreasing tendency was more striking. Increasing TTS content with corresponding rises in WMFP addition levels might be ascribed to the fact of enhanced LAB‐induced hydrolysis of sucrose into glucose. These results were in agreement with the findings of who evaluated the physicochemical properties of soy and milk solids' fortified low fat yogurt and reported negative effect of storage period on TSS content with TSS decreased in gradual manner as storages interval increased (Zanhi & Jideani, 2012). Moreover, LABs show better growth performance under temperature of 20–45°C and LABs perform optimally when fermentation is carried out at optimum temperature of 20°C for 24 h (Lim et al., 2019).

### 3.3 Effect on water‐holding capacity and syneresis

All WMFP‐supplemented (2%, 4%, and 6%) and control yogurt samples were evaluated in terms of their WHCs and syneresis. The results of WHC of yogurt samples are tabulated in Table 3. As evident from results of Table 4, WHC of control (*T*_0_) samples during all storage periods was ranged from 39.57% to 50.71%. WHC of 2% WMFP‐supplemented samples (*T*_1_) subjected to storage period from 1 to 28 days was found in range of 44.73%–52.37%. In case of 4% WMFP‐supplemented yogurt samples, the WHC was found to be in range of 46.51%–55.62% during all storage intervals spanning 1–28 days. In case of 6% WMFP‐supplemented yogurt samples, the WHC was 50.23%–58.61% during all storage intervals. Overall, all yogurt samples showed increasing trend in WHC with corresponding increases in addition levels of WMFP from 2% to 6%. On day 1, the WHC of supplemented samples ranged 52.37%–58.61%, whereas WHC levels on day 7, 14, 21, and 28 ranged 48.27%–57.23%, 45.23%–55.61%, 43.27%–53.64%, and 39.57%–50.23%, respectively. 6% WMFP‐supplemented samples showed the highest WHC (58.61 ± 0.46%). Until day 7, the WHC showed slightly decreasing tendencies as compared to WHC of day 1 samples. However, after day 7, all yogurt samples including control exhibited significant decline in WHC with corresponding increases in storage period from 14 to 28 days.

**TABLE 4:** Effect of white mulberry powder concentration and storage period on water‐holding capacity and syneresis of yogurt

Parameters	Storage period (day)	*T*_0_ (control)	*T*_1_ (2%)	*T*_2_ (4%)	*T*_3_ (6%)
Water‐holding Capacity (%)	1	50.70 ± 0.52^ef^	52.37 ± 0.41^cd^	55.60 ± 0.56^b^	58.60 ± 0.46^a^
	7	48.27 ± 0.38^h^	51.27 ± 0.54^def^	53.43 ± 0.81^c^	57.23 ± 0.18^a^
	14	45.23 ± 0.69^ij^	49.00 ± 0.47^gh^	51.70 ± 0.59^de^	55.60 ± 0.42^b^
	21	43.27 ± 0.50^k^	46.43 ± 0.39^i^	48.43 ± 0.70^h^	53.63 ± 0.38^c^
	28	39.57 ± 0.38^l^	44.73 ± 0.27^j^	46.50 ± 0.31^i^	50.23 ± 0.26f^g^
Syneresis (%)	1	25.63 ± 0.38^efg^	24.77 ± 0.52^fghi^	23.60 ± 0.46^ijk^	21.17 ± 0.26^l^
	7	26.33 ± 0.32^de^	25.60 ± 0.78^efgh^	24.37 ± 0.59^hij^	22.50 ± 0.25^k^
	14	27.80 ± 0.49^c^	26.77 ± 0.24^cde^	25.03 ± 0.56^fgh^	22.90 ± 0.12^k^
	21	29.43 ± 0.59^b^	27.73 ± 0.27^c^	25.90 ± 0.15^ef^	23.40 ± 0.25^jk^
	28	31.27 ± 0.26^a^	29.33 ± 0.57^b^	27.30 ± 0.50^cd^	24.63 ± 0.38^ghij^

*Notes*: Mean values are the result of three replications (*n* = 3). Means carrying the same small letters are significantly different from each other. *T*_0_: Control; *T*_1_: Sample fortified with 2% white mulberry powder concentration; *T*_2_: Sample fortified with 4% white mulberry powder concentration; *T*_3_: Sample fortified with 6% white mulberry powder concentration.

Syneresis is an indicative of the amount of serum or whey released from the WMFP‐supplemented and control yogurt samples. As evident from results of Table 4, syneresis of control (*T*_0_) samples during all storage periods was ranged from 25.63% to 31.27%. Syneresis of 2% WMFP‐supplemented samples (*T*_1_) subjected to storage period from 1 to 28 days was found in range of 24.77%–29.34%. In case of 4% WMFP‐supplemented yogurt samples, the syneresis was found to be in range of 23.60%–27.31% during all storage intervals spanning 1–28 days. In case of 6% WMFP‐supplemented yogurt samples, the syneresis was 21.17%–24.63% during all storage intervals. It was evident from the results that supplemented yogurt samples exhibited decreasing tendencies in syneresis with corresponding rise in addition levels of WMFP from 2% to 6%. Overall, all control samples showed significantly (*p* < .05) higher syneresis values in comparison with WMFP‐supplemented samples. Highest syneresis was recorded in control yogurt (28.09 ± 0.41%) (Table 4). One of the most influential factors is degree of denaturation of whey proteins which exert significant effect on yogurt texture and syneresis. Furthermore, incubation temperature also plays its role in terms of affecting syneresis. In this regard, lower temperature allows to take longer time for setting of curd whereas gradually increasing tendency in temperature causes increased syneresis in yogurt (Arab et al., 2022). Moreover, it has already been suggested in the published literature that dietary fiber of plant origin has absorptive role regarding absorption of serum or whey release from yogurt gel structure (Raza et al., 2021). The increasing WHC trend of yogurt with an increase in the concentration of powder might be due to an increase in TSS resulted because of the addition of WMFP. The decreasing trend in syneresis might be correlated to the fact of rises in WHC of mulberry fruit powder (Öztürk et al., 2018). This addition might have attributed to enhance the available carbohydrates, fiber, and pectic substances having high water‐binding ability (Singh & Muthukun, 2008). The results of the current study were in line with the results reported by Barkallah et al. (2017) whereby decreasing tendency in syneresis was brought about because of dietary fiber content in yogurt samples fortified with Spirulina as compared to control. Furthermore, the gradually decreasing trend in syneresis of supplemented yogurt samples was correlated to reduced serum release of whey which consequently led to improvement in water retention by yogurt samples owing to rising tendency in protein denaturation. As it was evident from results that WHC and syneresis are inversely interrelated to each other, hence, 4% and 6% WMFP‐supplemented yogurt samples exhibited higher WHC values as compared to 2% supplemented and control samples. Retention of high protein content and total solids in supplemented yogurt samples might be possibly the cause of rises in WHC. Previously published reports have reported similar results whereby high WHC was correlated to the recued degree of syneresis owing to high fiber content and total soluble solids (Jridi et al., 2015; Kermiche et al., 2018).

### 3.4 Effect on lactic acid bacterial count

**FIGURE 1:**
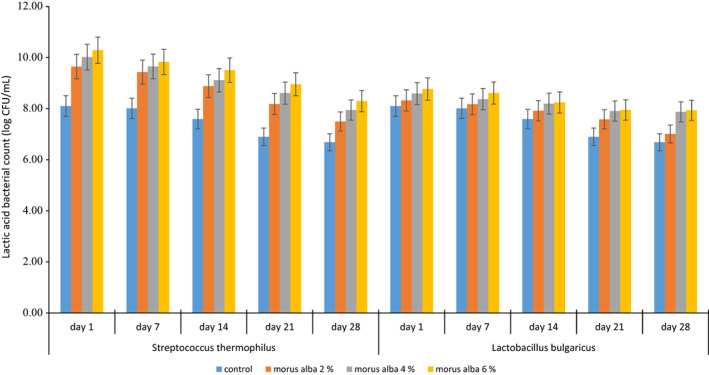
Viable bacterial count (*Streptococcus thermophilus* and *Lactobacillus bulgaricus*) in control and supplemented yogurts containing white mulberry fruit powder at various storage periods

The lactic acid bacteria (LABs) count (*S. thermophilus* and *L. bulgaricus*) during storage interval ranging from 1 to 28 days for control and WMFP‐supplemented samples are demonstrated in Figure 1. The LAB count of control samples during entire storage interval for *S. thermophilus* and *L. bulgaricus* ranged 8.10–10.29 and 8.01–8.61 log CFU/ml, respectively. Regardless of the storage period, all fortified yogurt samples exhibited decreasing trends in LAB count with corresponding increases in storage period. Initial *Lactobacillus bulgaricus* count was observed in range from 8.10 to 8.77 log CFU/ml which decreased to 6.68–7.94 log CFU/ml till the end of storage period. *Streptococcus thermophilus* showed the similar trend (Figure 1). Addition of white mulberry significantly (*p* < .05) increased the viable counts of *S. thermophilus* and *L. bulgaricus*. This increase might be attributed to the presence of polyphenols and fiber in food of a plant origin. Dietary fiber provided additional sources of carbohydrates and acts as fermentable substrate favoring lactic acid bacteria growth. Commonly, the viable counts of Streptococcus in yogurt is significantly greater than that of *Lactobacillus* (Zacarchenco & Massaguer‐Roig, 2006). A decrease in the viable count of yogurt bacteria during storage was reported by Cais‐Sokolińska and Pikul (2004). The yogurt sample added with 2% WMFP had significantly higher sensory values as compared to other samples (Table 5).

**TABLE 5:** Effect of white mulberry powder concentration and storage period on sensory evaluation of yogurt (*n* = 3)

	Storage period (day)	*T*_0_ (control)	*T*_1_ (2%)	*T*_2_ (4%)	*T*_3_ (6%)
Color	1	7.00 ± 0.00^abc^	7.67 ± 0.33^a^	6.67 ± 0.33^bcd^	6.00 ± 0.00^def^
	7	6.67 ± 0.33^bcd^	7.33 ± 0.33^ab^	6.33 ± 0.33^cde^	5.67 ± 0.33^efg^
	14	6.33 ± 0.33^cde^	7.00 ± 0.00^abc^	6.00 ± 0.00^def^	5.33 ± 0.33^fgh^
	21	5.67 ± 0.33^efg^	6.33 ± 0.33^cde^	5.67 ± 0.33^efg^	5.00 ± 0.00^gh^
	28	5.33 ± 0.33^fgh^	6.00 ± 0.00^def^	5.00 ± 0.00^gh^	4.67 ± 0.33^h^
Flavor	1	7.00 ± 0.00^a^	7.00 ± 0.00^a^	6.33 ± 0.33^abc^	6.00 ± 0.00^bcd^
	7	6.33 ± 0.33^abc^	6.67 ± 0.33^ab^	6.33 ± 0.33^abc^	5.67 ± 0.33^cde^
	14	6.00 ± 0.00^bcd^	6.33 ± 0.33^abc^	6.00 ± 0.00^bcd^	5.33 ± 0.33^def^
	21	5.67 ± 0.33^cde^	5.67 ± 0.33^cde^	5.33 ± 0.33^def^	5.00 ± 0.00^ef^
	28	5.00 ± 0.00^ef^	5.33 ± 0.33^def^	5.00 ± 0.00^ef^	4.67 ± 0.33^f^
Texture	1	6.67 ± 0.33^bcd^	7.67 ± 0.33^a^	7.33 ± 0.33^ab^	6.00 ± 0.00^def^
	7	6.33 ± 0.33^cde^	7.33 ± 0.33^ab^	7.00 ± 0.00^abc^	5.67 ± 0.33^efg^
	14	6.00 ± 0.00^def^	7.00 ± 0.00^abc^	6.33 ± 0.33^cde^	5.33 ± 0.33^fgh^
	21	5.33 ± 0.33^fgh^	6.33 ± 0.33^cde^	5.67 ± 0.33^efg^	5.00 ± 0.00^gh^
	28	5.00 ± 0.00^gh^	5.67 ± 0.33^efg^	5.00 ± 0.00^gh^	4.67 ± 0.33^h^
Sweetness	1	6.67 ± 0.33^a^	6.33 ± 0.33^ab^	6.33 ± 0.33^ab^	6.00 ± 0.00^abc^
	7	6.00 ± 0.00^abc^	6.00 ± 0.00^abc^	6.00 ± 0.00^abc^	5.67 ± 0.33^bcd^
	14	5.67 ± 0.33^bcd^	5.67 ± 0.33^bcd^	5.67 ± 0.33^bcd^	5.33 ± 0.33^cde^
	21	4.67 ± 0.33^ef^	5.00 ± 0.00^def^	5.33 ± 0.33^cde^	5.00 ± 0.00^def^
	28	4.33 ± 0.33^f^	4.33 ± 0.33^f^	5.00 ± 0.00^def^	4.33 ± 0.33^f^
Sourness	1	7.33 ± 0.33^a^	6.33 ± 0.33^bcd^	6.33 ± 0.33^bcd^	5.67 ± 0.33^def^
	7	7.00 ± 0.00^ab^	6.00 ± 0.00^cde^	5.67 ± 0.33^def^	5.00 ± 0.00^fgh^
	14	6.67 ± 0.33^abc^	5.67 ± 0.33^def^	5.33 ± 0.33^efg^	4.67 ± 0.33^gh^
	21	5.67 ± 0.33^def^	5.00 ± 0.00^fgh^	5.00 ± 0.58^fgh^	4.33 ± 0.33^hi^
	28	5.00 ± 0.00^fgh^	4.33 ± 0.33^hi^	4.33 ± 0.33^hi^	3.67 ± 0.33^i^
Taste	1	7.33 ± 0.33^ab^	7.67 ± 0.33^a^	7.33 ± 0.33^ab^	6.00 ± 0.00^def^
	7	7.00 ± 0.00^abc^	7.33 ± 0.33^ab^	7.00 ± 0.00^abc^	5.67 ± 0.33^efg^
	14	6.67 ± 0.33^bcd^	7.00 ± 0.00^abc^	6.67 ± 0.33^bcd^	5.33 ± 0.33^fgh^
	21	6.33 ± 0.33^cde^	6.33 ± 0.33^cde^	6.00 ± 0.00^def^	5.00 ± 0.00^gh^
	28	5.67 ± 0.33^efg^	5.67 ± 0.33^efg^	5.33 ± 0.33^fgh^	4.67 ± 0.33^h^
Overall acceptance	1	6.67 ± 0.33^bcd^	8.00 ± 0.00^a^	7.00 ± 0.00^bc^	6.00 ± 0.00^def^
	7	6.33 ± 0.33^cde^	7.33 ± 0.33^ab^	6.67 ± 0.33^bcd^	5.67 ± 0.33^ef^
	14	5.67 ± 0.33^ef^	7.00 ± 0.00^bc^	6.33 ± 0.33^cde^	5.33 ± 0.33^fg^
	21	5.33 ± 0.33^fg^	6.00 ± 0.58^def^	5.67 ± 0.33^ef^	4.67 ± 0.33^gh^
	28	4.33 ± 0.33^h^	5.67 ± 0.33^ef^	4.33 ± 0.33^h^	4.00 ± 0.00^h^

*Notes*: Mean values are the result of three replications (*n* = 3). Means carrying the same small letters are significantly different from each other. *T*_0_: Control; *T*_1_: Sample fortified with 2% white mulberry powder concentration; *T*_2_: Sample fortified with 4% white mulberry powder concentration; *T*_3_: Sample fortified with 6% white mulberry powder concentration.

The LAB count was considerably higher at start of the storage period (day 1) for all yogurt samples; however, the LAB count of both *S. thermophilus* and *L. bulgaricus* showed decreasing tendency by 1.5 log CFU/ml at the end of storage period (28th day). Similar results have been reported by Jaziri et al. (2009) who reported sufficiently higher microflora (8.27–9 log CFU/ml) in yogurt samples at beginning of 6‐week storage period. In food and feed industries, LABs are the most commonly employed probiotic for preparing functional foods and ingredients. Probiotic efficiency and survival is reliant on the strain's stability in gastrointestinal tract of host and, hence, the tolerance to the acid and bile is one of the key factors determining successful application probiotic strain. *M. alba* silage has been recorded to the potent source for LABs isolation. In a study carried out by Shokryazdan et al. (2015), about 38 LABs were isolated from the mulberry silage; however, only four strains were capable enough for survival in GIT tract. Mulberry pekmez has been reported to exhibit potential of lowering LAB count because of rises in the TSS and sugar content, and hence, it may be implied that LABs growth was significantly affected with corresponding rises in additive levels of mulberry fruit powder in fortified yogurts (Celik & Bakirci, 2003).

### 3.5 Effect on sensory attributes of supplemented yogurt

Sensory evaluation allows to record the preferences of human beings in terms of scores related to various sensory attributes, such as taste, texture, appearance, etc. Sensory evaluation was carried out for two types of yogurt samples, such as control and WMFP‐added yogurt samples (2%, 4%, and 6% supplemented samples). The color values for control samples during all storage periods ranged 5.33–7.01, whereas, for 2%, 4%, and 6% supplemented yogurt samples, the color values were observed in ranges of 6.01–7.67, 5.01–6.67, and 4.67–6.01, respectively. As evident from results of Table 5, color values of control (*T*_0_) samples during all storage periods were ranged from 5.33 to 7.01. Color values of 2% WMFP‐supplemented samples (*T*_1_) subjected to storage period from 1 to 28 days were found in range of 6.01–7.67. In case of 4% WMFP‐supplemented yogurt samples, the color values were found to be in range of 23.60%–27.31% during all storage intervals spanning 1–28 days. In case of 6% WMFP‐supplemented yogurt samples, the color values were ranged from 21.17% to 24.63% during all storage intervals. Overall, it was evident from the results that 2% addition levels of WMFP caused slight increases in color values of yogurt samples during all storage periods as compared to control, whereas further rises in addition levels led to decreasing tendencies in color values of supplemented yogurt samples in gradual manner. As evident from results of Table 5, flavor of control (*T*_0_) samples during all storage periods was ranged from 5.01 to 7.03. Flavor of 2% WMFP‐supplemented samples (*T*_1_) subjected to storage period from 1 to 28 days was found in range of 5.34–7.01. In case of 4% WMFP‐supplemented yogurt samples, the flavor was found to be in range of 5.01–6.34 during all storage intervals spanning 1–28 days. In case of 6% WMFP‐supplemented yogurt samples, the flavor was ranged 4.67–6.03 during all storage intervals. In case of flavor, 2% addition level led to slight improvement in flavor of yogurt samples as compared to control, whereas further increases in supplementation level up to 4% addition exhibited comparable flavor values as compared to control. However, 6% addition level did not cause any significant (*p* > .05) change in yogurt flavor scores; however, flavor scores varied slightly in decreasing manner. As evident from results of Table 5, texture of control (*T*_0_) samples during all storage periods was ranged from 5.01 to 6.67. Texture of 2% WMFP‐supplemented samples (*T*_1_) subjected to storage period from 1 to 28 days was found in range of 5.67–7.68. In case of 4% WMFP‐supplemented yogurt samples, the texture values were found to be in range of 5.01–7.34 during all storage intervals spanning 1–28 days. In case of 6% WMFP‐supplemented yogurt samples, the texture was ranged 4.67–6.03 during all storage intervals. Similarly, texture values for control, 2%, 4%, and 6% supplemented yogurt samples ranged 5.01–6.67, 5.67–7.68, 5.01–7.34, and 4.67–6.01, respectively. Overall, 2% addition level led to highest scores for texture as compared to control and other supplemented samples, whereas, 4% and 6% addition levels caused concentration‐dependent decreases in texture scores during all storage periods. This implied that 2% addition level was the most appropriate in retention of sensory properties of supplemented yogurt. Sweetness of control (*T*_0_) samples during all storage periods was ranged from 4.34 to 6.67. Sweetness of 2% WMFP‐supplemented samples (*T*_1_) subjected to storage period from 1 to 28 days was found in range of 4.32–6.35. In case of 4% WMFP‐supplemented yogurt samples, the sweetness was found to be in range of 5.01–6.34 during all storage intervals spanning 1–28 days. In case of 6% WMFP‐supplemented yogurt samples, the sweetness was ranged from 4.37–6.01 during all storage intervals. In case of sweetness score, the 2% and 4% additional levels did not cause any significant change in sweetness of supplemented yogurts and panelists scores remained invariably same; however, 6% addition levels led to slight decreases in sweetness of yogurt samples during all storage intervals except day 21. On 21th day, the 6% supplemented samples exhibit slightly higher sweetness score as compared to control. As presented in Table 5, sourness of control (*T*_0_) samples during all storage periods was ranged from 5.01 to 7.34. Sourness of 2% WMFP‐supplemented samples (*T*_1_) subjected to storage period from 1 to 28 days was found in range of 4.34–6.35. In case of 4% WMFP‐supplemented yogurt samples, the sourness was similar to those of 2% supplemented samples. In case of 6% WMFP‐supplemented yogurt samples, the sourness values were ranged 3.67–5.69 during all storage intervals. In case of sourness, the 2% and 4% addition levels exhibited invariably similar scores for sourness irrespective of the storage period as compared to control. However, further rises in WMFP caused slightly decreasing trends in sourness in comparison with control. Taste values of control (*T*_0_) samples during all storage periods were ranged from 5.67 to 7.34. Taste of 2% WMFP‐supplemented samples (*T*_1_) subjected to storage period from 1 to 28 days was found in range of 5.68–7.69. In case of 4% WMFP‐supplemented yogurt samples, the taste was found to be in range of 5.34–7.35 during all storage intervals spanning 1–28 days. In case of 6% WMFP‐supplemented yogurt samples, the taste values were ranged 4.67–6.01 during all storage intervals. Similarly, the taste attribute showed similar scores for 2% and 4% added yogurt samples as compared to that of control during all storage periods; however, 6% added samples showed slightly lower scores for taste attribute. As evident from results of Table 5, overall acceptance scores of control (*T*_0_) samples during all storage periods were ranged from 4.34 to 6.67. Overall acceptance scores of 2% WMFP‐supplemented samples (*T*_1_) subjected to storage period from 1 to 28 days were found in range of 5.67–8.01. In case of 4% WMFP‐supplemented yogurt samples, the overall acceptance scores were found to be in range of 4.34–7.03 during all storage intervals spanning 1–28 days. In case of 6% WMFP‐supplemented yogurt samples, the overall acceptance scores were ranged 4.01–6.05 during all storage intervals. Overall, all sensory attributes showed decreasing trends in scores with corresponding rises in storage periods from 1 to 28 days. Overall acceptability scores were also recorded by the panelists and results are tabulated in Table 5. The overall acceptability scores for 2%, 4%, and 6% supplemented samples ranged 5.67–8.01, 4.33–7.01, and 4.01–6.01, respectively, as compared to control (4.33–6.67). Overall, the highest overall acceptability scores were found in case of 2% supplemented yogurt samples as compared to control, whereas further rises in addition levels from 4% to 6% caused gradually decreasing tendencies in overall acceptance scores by the panelists. Moreover, it was also evident form the results that all samples including control showed declining trend in overall acceptance scores with corresponding increases in storage period from 1 to 28 days. Similarly, in a study carried out by Salih et al. (2020), whereby cow milk yogurt was evaluated for its sensory properties after addition of gum Arabic powder at range of 0.5%–1.5%. The authors reported that gum Arabic significantly (*p* < .05) affected the sensory properties of yogurt 0.5% addition level of gum Arabic powder exhibited the highest score of overall acceptability.

## 4 CONCLUSIONS

White mulberry has the potential to help in curing of diabetes mellitus, atherosclerosis, cardiovascular diseases, different neurological disorders, various skin infections as well as cancer. To best of our knowledge, yogurt added with white mulberry has never been studies so far. Therefore, the objective of this study was to investigate the effect of sun‐dried white mulberry fruit powder (WMFP) (2%, 4%, and 6%) for enhancing nutritional value and improving the quality of yogurt during refrigerated storage and evaluation of effects of white mulberry fruit powder (WMFP) on antioxidant activity, lactic acid bacterial count, physicochemical, and sensory attributes of yogurt. Results showed that the highest (*p* < .05) antioxidant activity of 54.53 **±** 0.15% was observed in 6% WMFP‐added yogurt samples. Highest pH of 4.53 ± 0.08 was observed in control. Significantly highest (*p* < .05) acidity (1.12 ± 0.02%) was recorded in the yogurt with 6% WMFP‐added yogurt samples on 28th day. Moreover, the addition of WMFP elevated the total solids up to 20.05 ± 0.02 °Brix and water‐holding capacity up to 55.06 ± 0.34% and lessened the syneresis value (22.92 ± 0.25**)** in 6% yogurt samples. 2% WMFP‐added yogurt sample was assigned the highest sensory score in terms of overall acceptability by the panelists (8.00 ± 0.00). The results demonstrated that the addition of WMFP significantly (*p* < .05) improved the antioxidant potential and quality of yogurt. Thus, it was concluded that fruit powder of white mulberry can be used to enhance antioxidant potential, physicochemical, and sensory properties of fortified yogurt. This addition in yogurt enhanced its antioxidant potential and physicochemical quality. The study introduces white mulberry‐enriched yogurt and suggests the food industries to launch this product in the market. Therefore, white mulberry fruit powder can be incorporated in yogurt for elevating nutritional value, improving yogurt quality, and for enhancement of yogurt functionality.

## CONFLICT OF INTEREST

Authors have no conflict of interest to declare.

## Data Availability

The data used to support the findings of this study are available from the corresponding author upon request.
